# External and circadian inputs modulate synaptic protein expression in the visual system of *Drosophila melanogaster*

**DOI:** 10.3389/fphys.2014.00102

**Published:** 2014-04-03

**Authors:** Wojciech Krzeptowski, Jolanta Górska-Andrzejak, Ewelina Kijak, Alicja Görlich, Elżbieta Guzik, Gareth Moore, Elżbieta M. Pyza

**Affiliations:** Department of Cell Biology and Imaging, Institute of Zoology, Jagiellonian UniversityKraków, Poland

**Keywords:** synaptic plasticity, circadian rhythms, light stimulation, locomotor activity, the first optic neuropil

## Abstract

In the visual system of *Drosophila melanogaster* the retina photoreceptors form tetrad synapses with the first order interneurons, amacrine cells and glial cells in the first optic neuropil (lamina), in order to transmit photic and visual information to the brain. Using the specific antibodies against synaptic proteins; Bruchpilot (BRP), Synapsin (SYN), and Disc Large (DLG), the synapses in the distal lamina were specifically labeled. Then their abundance was measured as immunofluorescence intensity in flies held in light/dark (LD 12:12), constant darkness (DD), and after locomotor and light stimulation. Moreover, the levels of proteins (SYN and DLG), and mRNAs of the *brp*, *syn*, and *dlg* genes, were measured in the fly's head and brain, respectively. In the head we did not detect SYN and DLG oscillations. We found, however, that in the lamina, DLG oscillates in LD 12:12 and DD but SYN cycles only in DD. The abundance of all synaptic proteins was also changed in the lamina after locomotor and light stimulation. One hour locomotor stimulations at different time points in LD 12:12 affected the pattern of the daily rhythm of synaptic proteins. In turn, light stimulations in DD increased the level of all proteins studied. In the case of SYN, however, this effect was observed only after a short light pulse (15 min). In contrast to proteins studied in the lamina, the mRNA of *brp*, *syn*, and *dlg* genes in the brain was not cycling in LD 12:12 and DD, except the mRNA of *dlg* in LD 12:12. Our earlier results and obtained in the present study showed that the abundance of BRP, SYN and DLG in the distal lamina, at the tetrad synapses, is regulated by light and a circadian clock while locomotor stimulation affects their daily pattern of expression. The observed changes in the level of synaptic markers reflect the circadian plasticity of tetrad synapses regulated by the circadian clock and external inputs, both specific and unspecific for the visual system.

## Introduction

In the first optic neuropil (lamina) of the fly's visual system the majority of synaptic contacts is formed between the terminals of the compound eye photoreceptors and their postsynaptic partners. They are called tetrad synapses and use histamine (Hardie, 1987; Melzig et al., 1998; Gavin et al., 2007) to transmit photic and visual information to four lamina postsynaptic cells: L1 and L2 monopolar cells, amacrine cell, and glial or L3 monopolar cell (Meinertzhagen, 1989; Meinertzhagen and Sorra, 2001). Counting the presynaptic profiles of these synapses (the so-called T-bars) at the electron microscope level revealed that their number oscillates during the 24 h day/night cycle in both *Musca domestica* (Pyza and Meinertzhagen, 1993) and *Drosophila melanogaster* (Woźnicka and Pyza, unpublished data). In the photoreceptors of *D. melanogaster* the number of tetrad's T-bars increases two times during the day: at the beginning of the day and at the beginning of the night (Woźnicka and Pyza, unpublished data). This pattern of changes resembles the rhythm of locomotor activity of the fruit fly. Recently, we also found that a similar pattern of changes is characteristic for daily alterations in the level of synaptic protein Bruchpilot (BRP) in the lamina and in the whole brain of *D. melanogaster* (Górska-Andrzejak et al., 2013). BRP is a presynaptic active zone protein, homologous to the mammalian ELKS/CAST family of synaptic proteins (Wagh et al., 2006) that facilitates efficient vesicle release (Kittel et al., 2006). In *D. melanogaster* it has been detected in dense bodies of neuromuscular junctions (NMJ) (Wagh et al., 2006; Fouquet et al., 2009) and at the lamina T-bar synapses (Górska-Andrzejak et al., 2009a; Górska-Andrzejak et al., 2013; Hamanaka and Meinertzhagen, 2010).

So far, BRP is the only synaptic protein whose level of expression in the lamina and the whole head has been found to oscillate in a circadian manner (Górska-Andrzejak et al., 2013). However, it is possible that the amount of other key proteins of the synaptic machinery may also be influenced by the circadian system and/or by various internal and external factors. Another presynaptic protein (although not the structural one like BRP) is Synapsin (SYN), an evolutionary conserved phosphoprotein associated with synaptic vesicles (Hilfiker et al., 1999; Südhof, 2004; Evergren et al., 2007). In *D. melanogaster*, as well as in vertebrates, SYN has been shown to maintain vesicle release by recruiting synaptic vesicles from the reserve pool (Li et al., 1995; Pieribone et al., 1995; Sun et al., 2006; Akbergenova and Bykhovskaia, 2007).

The synaptic protein reported to be important for the synaptic assembly and plasticity is Discs large (DLG), a member of the MAGUK (Membrane-Associated Guanylate Kinase) family of scaffolding proteins. DLG has four mammalian members; SAP97/hDlg, PSD-93/Chapsyn-110, PSD-95/SAP90, and SAP102/NE-Dlg, which play important roles in the development and regulation of the nervous system and epithelia (Wang et al., 2011). In *D. melanogaster* DLG is involved in the development of epithelia (Woods et al., 1996), asymmetric cell division (Ohshiro et al., 2000), the development and plasticity of the larval NMJ (Lahey et al., 1994; Budnik et al., 1996; Guan et al., 1996; Tejedor et al., 1997; Thomas et al., 1997), as well as in other synaptic functions that are important for behavior of adult flies, including their circadian rhythms (Mendoza-Topaz et al., 2008). DLG is thought to play a key role in clustering GluRIIB receptors at the NMJ synapses and Shaker K^+^ channels throughout the CNS (Chen et al., 2008). In the brain of *D. melanogaster* DLG is highly expressed in the visual system (Rogero et al., 1997; Ruiz-Canada et al., 2002), while in the lamina it has been localized in the membranes of photoreceptor terminals that surround the invaginating heads of the glial capitate projections (Hamanaka and Meinertzhagen, 2010). At NMJs, DLG is highly concentrated postsynaptically, in the subsynaptic reticulum (SSR) and it is less prominently accumulated at the presynaptic membrane (Guan et al., 1996). However, DLG expression is important for the structure of both pre- and postsynaptic sites, since in *dlg* mutants, that survive only to the pupal stage, the number of NMJ T-bars is increased and SSR is reduced (Lahey et al., 1994).

In the present study we examined circadian changes in the level of three crucial synaptic proteins; BRP, SYN, and DLG, and their gene expression in order to learn about their possible involvement in the circadian synaptic plasticity of tetrad synapses in the lamina and in the brain. In addition we analyzed their responses to external factors, such as direct light exposure and locomotor stimulation.

## Materials and methods

### Animals

All experiments were conducted on a wild type Canton S strain of *Drosophila melanogaster*. The stock was maintained on a standard yeast-cornmeal-agar medium at 25 ± 1°C, under a day/night cycle (12 h of light and 12 h of darkness; LD 12:12). Following their eclosion, the experimental flies were kept in LD 12:12 for 1 week and the 7 days old males were decapitated at the specific time points of day and night. These time points were chosen to reflect significant features of the circadian locomotor activity pattern. To examine the expression of studied genes and proteins during the morning and evening locomotor activity peaks, we chose ZT1 and ZT12 or ZT13 (ZT—Zeitgeber Time; ZT0—the beginning of the day, ZT12—the beginning of the night), respectively. The afternoon siesta was examined at ZT4 or ZT6 and sleep at ZT16 or ZT18. To determine the role of the circadian clock in regulating the studied rhythms, another group of the experimental flies was transferred to constant darkness (DD) after 4 days in LD 12:12. On the third day of DD, flies were decapitated at CT1, CT4 or CT6 and CT12 or CT13, and CT16 or CT18 (CT—Circadian Time; CT0—the beginning of the subjective day, CT12—the beginning of the subjective night).

Decapitations of flies in the dark phase of the LD 12:12, as well as all flies kept in DD were conducted under dim red lighting (Pyza and Meinertzhagen, 1996).

### Locomotor and light stimulations

For locomotor stimulation experiments, all flies were kept in empty 250 ml Erlenmeyer flasks. One hour prior to decapitation the experimental flies were forced to fly by shaking the flask. For light stimulation experiments, flies were kept in LD 12:12 for 4 days before being transferred to DD. On the third day in conditions of constant darkness they were stimulated with a white light pulse for either 15 or 60 min before their decapitation at CT1. Control groups were kept in constant darkness.

### Immunohistochemistry

The heads of the flies collected at the experimental time points (ZTs or CTs) were processed in parallel, under the same conditions. Flies were immobilized with CO_2_ and decapitated in a drop of fixative—4% formaldehyde (PFA) in a 0.1 M phosphate buffer (PB). Following tissue fixation and overnight infiltration in a 25% solution of sucrose, the cryostat sections of heads were cut and incubated with one of the three mouse monoclonal antibodies: nc82, 3C11, or 4F3 [Developmental Studies Hybridoma Bank (DSHB)] detecting the synaptic proteins BRP, SYN, and Disc Large (DLG), respectively (Kittel et al., 2006; Wagh et al., 2006; Hofbauer et al., 2009; Hamanaka and Meinertzhagen, 2010). The 4F3 antibody was produced against the PSD-95/DLG/zona occludens-1 2 (PDZ-2) (Parnas et al., 2001). After several washes in a 0.01 M sodium phosphate buffer (PBS) containing 0.2% Triton-X (Sigma), the tissue was incubated with the goat anti-mouse secondary antibody conjugated with Cy3 (Jackson ImmunoResearch Laboratories Inc.). Sections were examined using a Zeiss LSM 510 Meta confocal microscope following washing and mounting in a Vectashield medium (Vector).

### Quantification of immunolabeling

**Figure 1:**
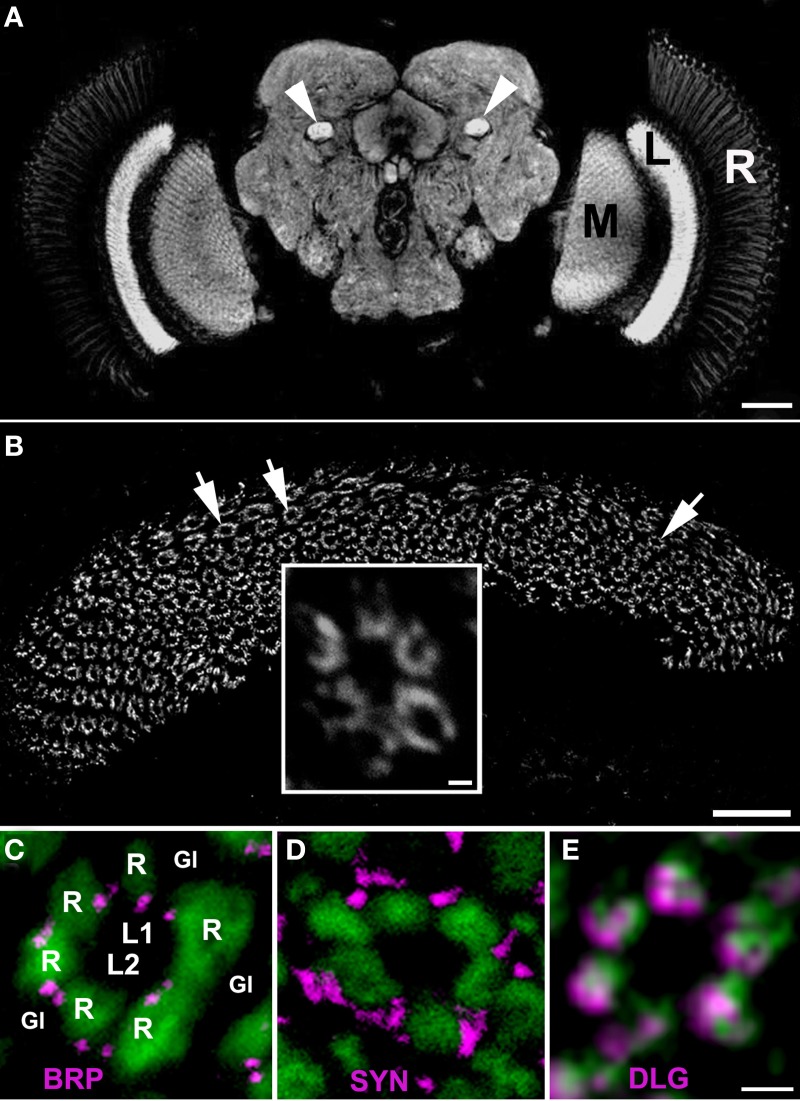
**(A)** The synaptic neuropils of the lamina (L), medulla (M) and the central brain of *Drosophila melanogaster* visualized by anti–BRP immunostaining using Mab nc82. There is a strong fluorescent signal in the mushroom body pedunculi in the central brain as well (arrow heads). R, retina. Scale bar: 50 μm. **(B)** The array of cartridges (arrows) in the cross sectioned neuropil of lamina as revealed by immunostaining using Mab 4F3 against DLG. Scale bar: 20 μm. Insert: the magnification of a single cartridge. Scale bar: 1 μm. **(C–E)** Cross sections of cartridges of GMR-Gal4 × UAS-S65T-GFP transgenic flies showing targeted expression of a green fluorescent protein (GFP) in photoreceptors (green) and immunolabeling (magenta) with Mab nc82 against BRP **(C)**, 3C11 against SYN **(D)** and 4F3 against DLG **(E)**. R—the terminals of six photoreceptors, L1, L2—interneurons receiving photic and visual information from photoreceptors, the so-called large monopolar cells (LMCs), Gl—the epithelial glial cells that surround lamina cartridges. Scale bar: 1 μm.

In each experiment, the images of the lamina of flies decapitated at different time points were collected at non-saturated settings, using identical image acquisition parameters. The changes in the level of fluorescence of BRP, SYN, and DLG-specific immunolabeling (brightness), corresponding to the changes in the amount of the given protein (BRP, SYN, or DLG), were quantified using ImageJ software (NIH). In each experiment the intensity of fluorescence was measured in the cartridges of the distal part of the cross-sectioned lamina (the second and the third row of cartridges from the lamina cortex) (Figure 1B), as represented by the Mean Gray Value, defined by the sum of the gray values of all pixels in the selected area, divided by the number of pixels within the selection. In the ImageJ software, the range of gray values in 8-bit images is divided into 256 bins. For each time point (ZTs or CTs) we collected data from 7 to 12 individuals. The background signal was subtracted.

### Determination of mRNA

For brain dissection we used the method described by Fujita et al. (1987). To summarize, experimental flies, some 15–20 individuals per time point, were frozen in liquid nitrogen. Subsequently, flies were transferred into glass tubes filled with 2 ml of cold acetone (−80°C) for dehydration, and stored at −80°C for 1 week. During that time, the acetone was replaced twice. Then, flies brains were dissected and total RNA was isolated using a NucleoSpin RNA XS kit (Macherey-Nagel) according to the manufacturer's protocol. To avoid contamination by genomic DNA, samples were treated with rDNase. RNA was eluted in 11 μl of RNase-free water. Reverse transcription was performed according to the manufacturer's protocol using a SuperScript III First-Strand Synthesis System for RT-PCR kit (Invitrogen), for *dlg* analysis or a High Capacity cDNA Reverse Transcription Kit (Applied Biosystems) for analysis of *brp* and *syn*. cDNA was prepared in 20 μl volumes using an oligo(dT)_20_ primer or random primers. Gene quantification was performed using the Step One Plus Real-Time PCR System (Applied Biosystems) and TaqMan Gene Expression Assays (Applied Biosystems). *dlg* (Assay ID: Dm01799278_g1), *brp* (Assay ID: Dm0194336_g1), and *syn* (Assay ID: Dm02148572_m1) genes were examined. Quantitative PCR was performed with 1 μl of cDNA as a template in a final reaction volume of 20 μl. Thermal cycling conditions were as follows: 2 min at 50°C, 10 min at 95°C followed by 40 repeats of 15 s at 95°C, and 1 min at 60°C. Data collection was performed during each annealing phase. Raw C_*T*_ values were collected and analysis was performed according to the 2^−ΔΔCT^ method, using *rpl32* (Assay ID: Dm02151827_g1) as a reference gene. Gene quantifications were completed using the software supplied by, and according to the instructions of the manufacturer. All experiments were performed independently at least three times, and each experiment comprised three parallel samples. In each run a negative control (distilled water instead of cDNA) was included.

### Western blot analysis

Flies collected at different time points (ZTs and CTs) were immediately frozen in liquid nitrogen. Their heads (20–30 heads per time point) were cut off, frozen and homogenized by sonification in a RIPA buffer (1.5 μl/1 head) containing a complete protease inhibitor cocktail (Boehringer). After being gently shaken for 1 h on ice, the obtained homogenates were stored at −20°C until centrifugation. Samples were centrifuged at 13,200 rpm for 1 h at 4°C and supplemented with a 2 × Laemmli buffer (1:1) before denaturation at 100°C for 5 min. The protein level was measured with a Quant-iT Protein Assay Kit using a Qubit fluorometer (Invitrogen). Protein extracts were subjected to electrophoresis using the NuPAGE SDS-PAGE Gel System. The 4–12% Bis-Tris gel was loaded with 20 or 10 μg (SYN and DLG, respectively) of the total protein per lane. The proteins were blotted by electrotransfer onto a PVDF membrane that was blocked with 5% nonfat dry milk in PBS with 0.1% Tween-20. Following blocking, the membrane was immunoprobed either with Mab 3C11 (1:10,000) or Mab 4F3 (1:20,000). The effectiveness of protein loading (loading control) was defined by probing the blots with the mouse monoclonal anti-α tubulin antibody (AA4.3), diluted 1:20,000 (DSHB). The ECL detection system (Perkin Elmer Inc.) was applied for immunodetection, whereas the AlphaEaseFC Stand Alone image analysis program (Alpha Innotech, Cell Bioscience,) was used for the densitometric analysis of the obtained Western blots. The collected data derived from three independent experiments providing three distinct protein preparations.

### Statistics

All statistical tests were performed using the Statistica v.10 (StatSoft, Inc.). The significance of differences observed between two independent groups (e.g., day and night) were statistically analyzed using the *T*-test or Mann–Whitney Test, depending on the result of the Shapiro–Wilk *W*-Test. Statistical significance across groups, ZT or CT time points, was analyzed using One-Way ANOVA followed by a Tukey *post-hoc* Test or the nonparametric counterpart of ANOVA—Kruskal–Wallis Test (one-way test), followed by the Multiple Comparison Test. To assess differences between LD 12:12 and DD we performed the Two-Way ANOVA regarding the light conditions and the time points (ZTs and CTs) as two key factors.

Immunolabeling quantification data are shown as a percentage of the highest value (100%) that was obtained in a given experiment (means ± SD).

For visualization of qPCR data, gene expression was normalized on an arbitrary scale, where the ZT1 time point was set to 1.00 (means ± SE).

## Results

The patterns of immunolabeling of BRP, SYN, and DLG in the whole brain of *D. melanogaster* was similar, if not identical. In each case, the immunofluorescence was confined to neuropils of the visual system and the central brain, as displayed by an exemplary image of BRP–specific immunolabeling in Figure 1A. Analysis of these labelings in the cross sectioned lamina showed that each of the studied proteins is concentrated within the synaptic units or cartridges, revealing the array of cartridges that constitute this neuropil (Figure 1B). However, the pattern of expression within the cartridge (Figures 1C–E) is different for each of these proteins, forming well defined spots in case of BRP (which localizes to T-bar ribbons) and being more dispersed in the case of SYN or DLG (Figures 1C–E).

### Bruchpilot

**Figure 2:**
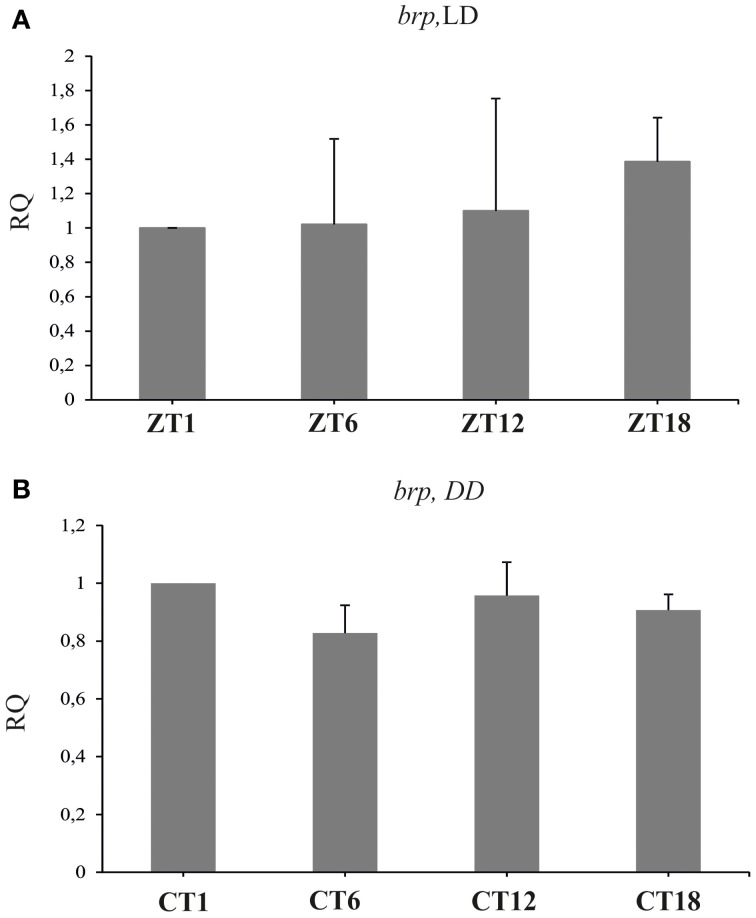
**The relative level of *brp* mRNA in the brain of flies raised in LD 12:12 (A) and in constant darkness (B)**. The level of *brp* mRNA is constant during the day and night in LD and DD.

The *brp* mRNA did not cycle in the brain of flies raised either in LD 12:12 (Figure 2A) [Kruskal–Wallis Test: *H*_(3, *N* = 12)_ = 2.63, *p* = 0.453] or in DD (Figure 2B) [Kruskal–Wallis Test: *H*_(3, *N* = 12)_ = 3.04, *p* = 0.38].

**Figure 3:**
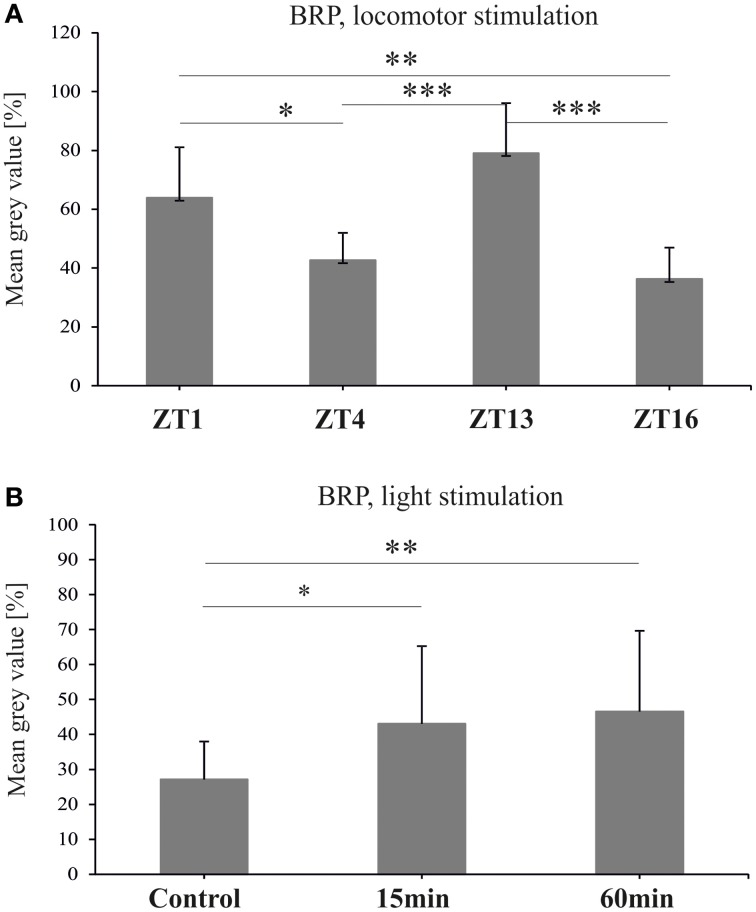
**The level of BRP-specific immunofluorescence intensity (brightness) in the fly's lamina after locomotor (A) and light (B) stimulations**. Both stimulations increase BRP level (^*^p ≤ 0.05, ^**^p ≤ 0.01, ^***^p ≤ 0.001).

After 1 h locomotor stimulation, the level of BRP fluctuated (ANOVA, *p* < 0.0001), being higher at ZT1 and ZT13 in comparison to ZT4 and ZT16 (Figure 3A) (Tukey's HSD Test, differences between ZT1 and ZT4 at *p* < 0.05; between ZT1 and ZT16 at *p* < 0.01; between ZT4 and ZT13 at *p* < 0.001, and between ZT13 and ZT16 at *p* < 0.001). However, the amplitude of the observed changes was larger than in non-stimulated flies (Górska-Andrzejak et al., 2013).

Light stimulation profoundly increased the level of BRP in the lamina of *D. melanogaster* [Kruskal–Wallis Test: *H*_(2, *N* = 57)_ = 11.08, *p* = 0.004] (Figure 3B). When compared to the non-stimulated controls, the intensity of BRP-specific immunofluorescence in the lamina of flies exposed to the light pulse at CT1 for either 15, or 60 min, increased by 58 and 63%, respectively (Multiple Comparison Test, *p* = 0.02 and *p* = 0.005, respectively).

### Synapsin

**Figure 4:**
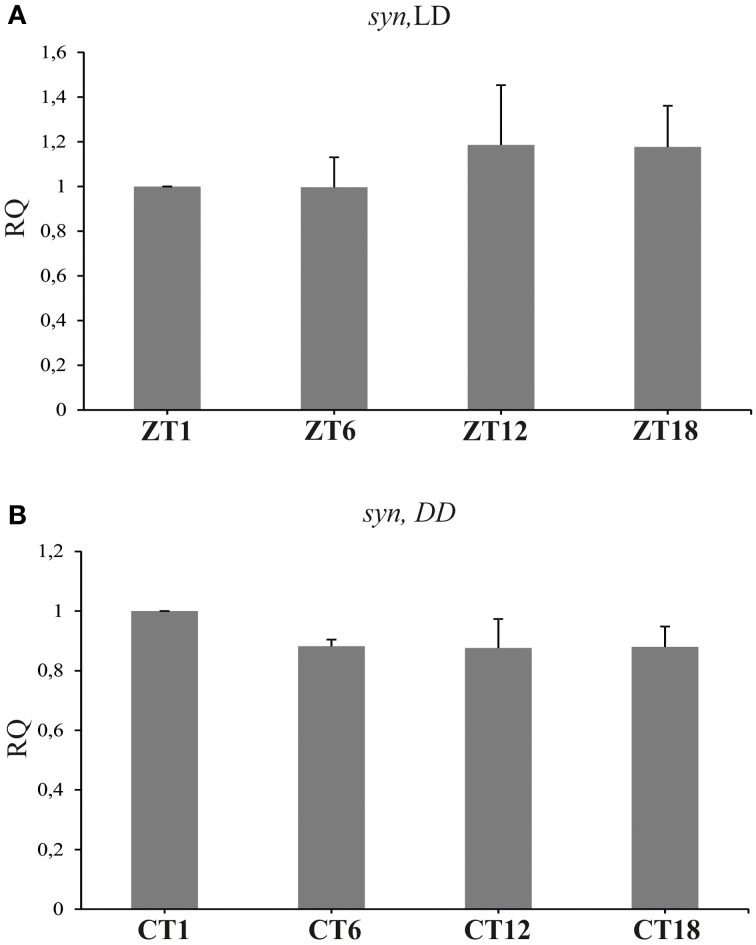
**The relative level of *syn* mRNA in the brain of flies raised in LD 12:12 (A) and in constant darkness (B)**. The mRNA of *syn* does not cycle in LD 12:12 or in DD.

The level of *syn* mRNA did not cycle in the brain of flies held either in LD 12:12 [Kruskal–Wallis Test: *H*_(3, *N* = 12)_ = 1.37, *p* = 0.7], or in DD [Kruskal–Wallis Test: *H*_(3, *N* = 12)_ = 3.82, *p* = 0.3] (Figures 4A,B). Also the total amount of SYN in whole head homogenates studied using the Western blotting method, did not change significantly during the 24 h period, either in LD 12:12 (ANOVA, *F* = 0.18, *p* = 0.9) or in DD [Kruskal–Wallis Test *H*_(3, *N* = 12)_ = 5.1, *p* = 0.16] (data not shown).

**Figure 5:**
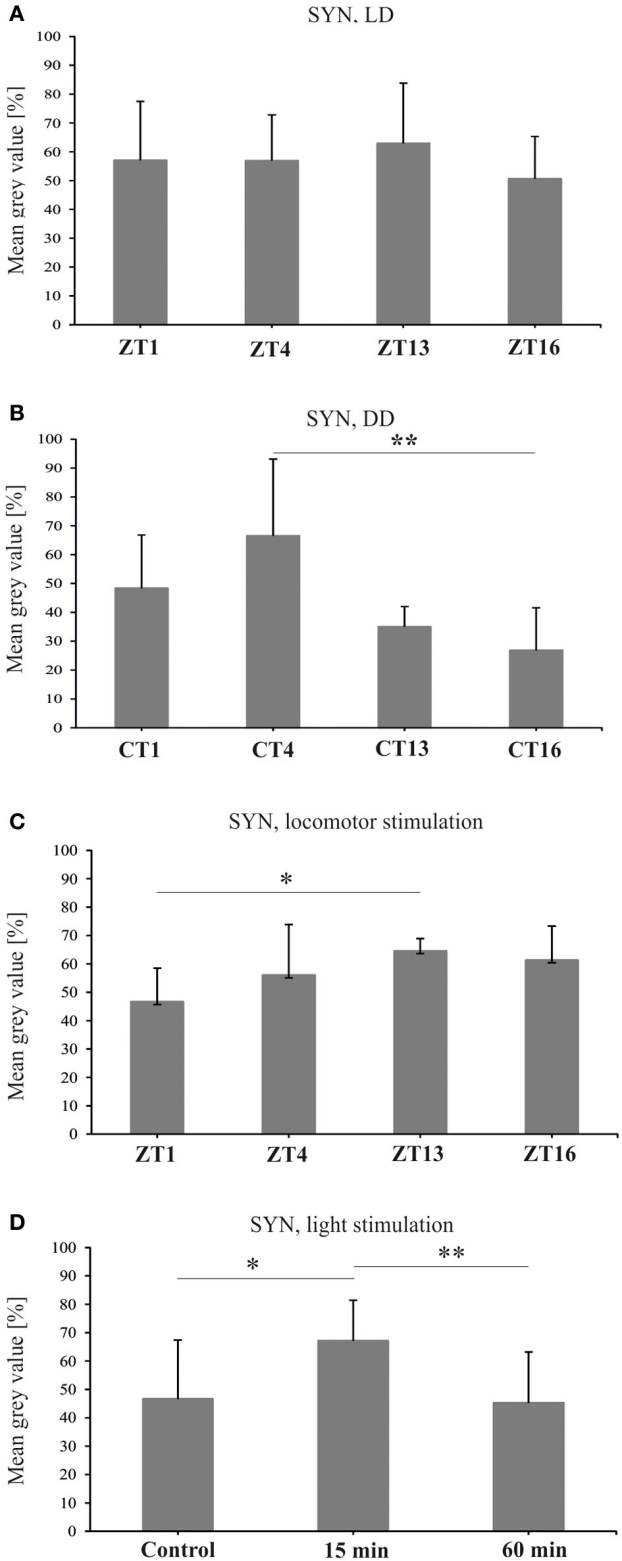
**Synapsin-specific immunofluorescence (brightness) measured from confocal images of the fly's lamina**. The level of this protein is the same during the day and night of LD 12:12 **(A)**, oscillates in constant darkness **(B)**, changes after 1 h of locomotor stimulation **(C)** and increases after 15 min of light stimulation **(D)** (^*^p ≤ 0.05, ^**^p ≤ 0.01).

Similar results were obtained for SYN protein in the lamina of flies held in LD 12:12. The analysis of the fluorescence intensity of immunolabeled SYN showed that the abundance of this protein did not change significantly in the course of day and night in LD 12:12 (ANOVA, *F* = 0.54, *p* = 0.7) (Figure 5A). The average level of SYN-specific fluorescence during the day and the night was the same (*t*-test, *t* = 0.02, *df* = 26, *p* < 0.98). In DD, on the other hand, there were significant changes in the level of SYN-immunospecific fluorescence in the lamina over the 24 h period [Kruskal–Wallis Test (3, *N* = 31) = 14.7, *p* = 0.002] (Figure 4B). The fluorescence intensity was the highest in the middle of the subjective day, at CT4, and the lowest in the middle of the subjective night, at CT16 (Figure 5B). The difference between these two time points was statistically significant (Multiple Comparison Test, *p* = 0.002). The average level of fluorescence during the subjective day was the same as during the day in LD 12:12, but was 31% lower during the subjective night, than during the night in LD 12:12. The difference between the subjective day and the subjective night was statistically significant (Mann–Whitney Test, *U* = 32, *p* = 0.0005) and so was the difference between the daily patterns of SYN-specific immunofluorescence in DD and LD 12:12 (MANOVA, *F* = 3.2, *p* = 0.03). Moreover, the average level of fluorescence in DD was 22% lower than in LD 12:12 (Mann–Whitney Test, *U* = 254.5, *p* = 0.007).

After 1 h locomotor activity stimulation at different times of the day, the intensity of SYN-specific immunofluorescence in the lamina displayed significant changes in the course of the day [Kruskal–Wallis Test (4, *N* = 53) = 18, *p* = 0.001] (Figure 5C). It was the lowest in the lamina of flies stimulated at the beginning of the day (ZT1—47%). At that time the fluorescence intensity was considerably lower (Multiple Comparison Test, *p* = 0.02) than at the beginning of the night (ZT13), when it reached the highest value measured for this set of data (65%). Also the average level of fluorescence during the day (ZT1 and ZT4) was significantly lower (11%) than during the night (ZT13 and ZT16) (Mann–Whitney Test, *U* = 88, *p* = 0.001).

After light stimulation at CT1 the level of SYN in the lamina increased but only after the short 15 min light pulse [Kruskal–Wallis Test (2, *N* = 35) = 11.05, *p* = 0.004] (Figure 5D). The short pulse increased SYN-specific immunofluorescence intensity by 21% when compared with the control. However, after a longer, 60 min light pulse, the level of SYN was similar to the control. The differences between the 15 min light pulse and the control (67 vs. 47%), as well as between the 60 min and 15 min pulses (67 vs. 45%), were significant (Multiple Comparison Test, *p* = 0.02 and *p* = 0.006, respectively).

### Disc large

**Figure 6:**
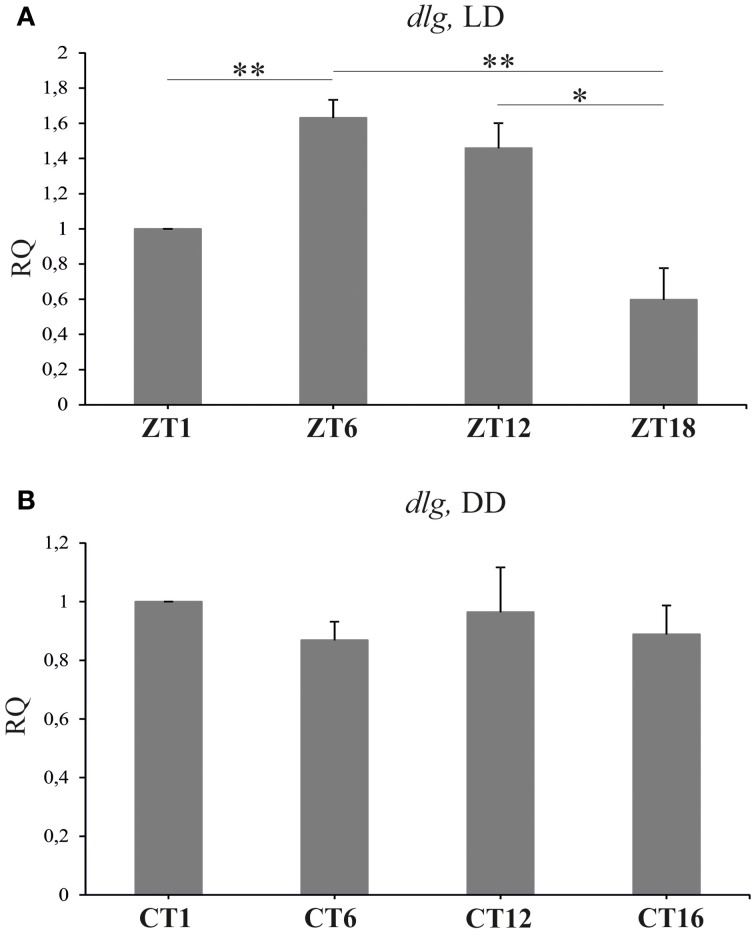
**The relative level of *dlg* mRNA in the brain of flies held in LD 12:12 or DD**. In LD 12:12 the mRNA of *dlg* changes during 24 h cycle with a peak in the middle of the day (ZT6) and a trough in the middle of the night **(A)**. No rhythm was observed in DD **(B)**; ^*^p ≤ 0.05, ^**^p ≤ 0.01.

Real-time PCR quantification revealed significant changes in the expression of *dlg* in the brain of *D. melanogaster* collected at different time points of the LD 12:12 cycle [Kruskal–Wallis Test: *H*_(3, *N* = 23)_ = 17.7, *p* = 0.0005] (Figure 6A). The highest level of *dlg* mRNA was detected in the middle of the day (ZT6) and at the end of the day (ZT12), whereas the lowest was typical for the end of the night (ZT18). The differences between ZT1 and ZT6 (63%) or ZT1 and ZT18 (103%) were statistically significant (Multiple Comparison Test, *p* = 0.01 and *p* = 0.005 respectively), as was the difference between ZT12 and ZT18 (86%, Multiple Comparison Test, *p* = 0.05). The expression of *dlg* in the brain of flies raised in constant conditions of darkness did not change during the subjective day and night [Kruskal–Wallis Test: *H*_(3, *N* = 28)_ = 4.83, *p* = 0.18] (Figure 6B). In turn, the total amount of DLG in the whole head homogenates obtained from flies kept in LD 12:12 or DD, was the same during the 24 h cycle [Kruskal–Wallis Test (3, *N* = 12) = 1.17, p = 0.76; Kruskal–Wallis Test (3, *N* = 8) = 1.13, *p* = 0.72, respectively] (data not shown).

**Figure 7:**
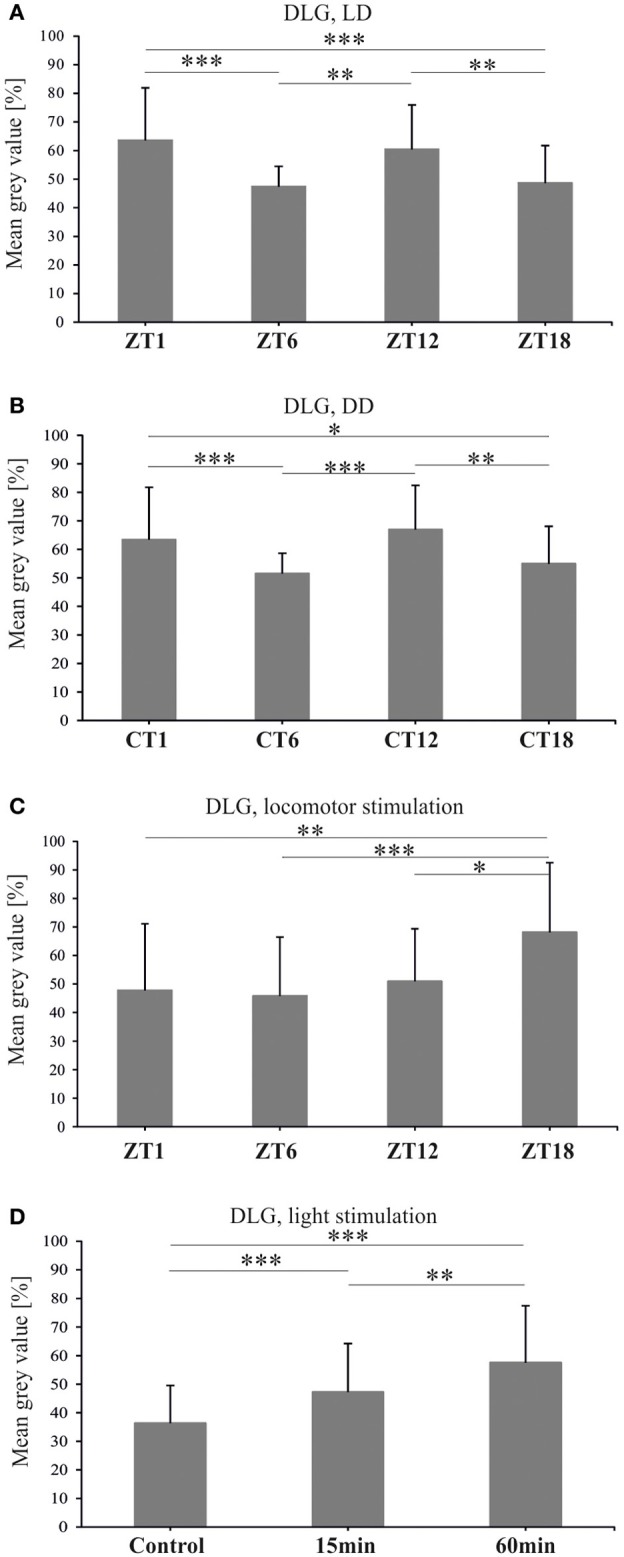
**DLG-specific immunofluorescence (brightness) measured from confocal images of the fly's lamina**. In both LD 12:12 **(A)** and DD **(B)** the level of DLG oscillates and its pattern is similar in both conditions with two peaks at ZT1 or CT1 and ZT12 or CT12. One hour of locomotor stimulation changes the rhythm of DLG **(C)**. Light stimulation increases DLG abundance in the lamina of wild type flies **(D)** (^*^p ≤ 0.05, ^**^p ≤ 0.01, ^***^p ≤ 0.001).

The abundance of DLG in the distal lamina of flies held in LD 12:12, showed significant changes during the light/dark cycle [Kruskal–Wallis Test: *H*_(3, *N* = 174)_ = 34.2, *p* < 0.0001] (Figure 7A). The pattern of changes in the DLG level in the lamina was bimodal with two peaks, at ZT1 and ZT12, as in the case of BRP (Górska-Andrzejak et al., 2013). The average intensity of DLG-specific fluorescence at these two time points of the cycle was the same (Multiple Comparison Test: *p* < 0.1), whereas it was lower in the middle of the day (ZT6) and in the middle of the night (ZT18). The differences between ZT1 and ZT6 (16%) or ZT1 and ZT18 (15%) were statistically significant (Multiple Comparison Test: *p* < 0.0001 and *p* < 0.0001, respectively), as well as the differences detected between ZT6 and ZT12 (13%, Multiple Comparison Test: *p* = 0.002) or ZT12 and ZT18 (12%, Multiple Comparison Test: *p* = 0.006). Similar oscillations in the level of DLG-specific immunofluorescence were also observed in the distal lamina of flies kept in constant darkness [Kruskal–Wallis Test: *H*_(3, *N* = 198)_= 27.3, *p* < 0.0001] (Figure 7B). Interestingly, the circadian pattern of changes was the same as the one observed in LD 12:12, since the fluorescence intensity was significantly higher at CT1 and CT12 than at CT4 and CT18. The differences between CT1 and CT6 (12%) or CT18 (8%), although slightly smaller than between ZT1 and ZT6 or ZT16, were statistically significant (Multiple Comparison Test: *p* = 0.0008 and *p* = 0.04, respectively). Also the differences between the second peak of DLG abundance at CT12 and CT6 (15%) or CT18 (12%) were significant (Multiple Comparison Test: *p* < 0.0001 and *p* = 0.004, respectively).

The above described pattern of DLG rhythm in the lamina of flies kept in LD 12:12 was changed by locomotor stimulation (Figure 7C). After a 1 h long locomotor stimulation of flies, administered prior to their decapitation, the intensity of DLG-specific fluorescence was still fluctuating during the 24 h cycle [Kruskal–Wallis Test: *H*_(3, *N* = 149)_ = 24.8, *p* < 0.0001]. However, the daily pattern was altered with a peak at the end of the night (ZT18) when the fluorescence level was 20, 22, and 17% more intensive than at ZT1, ZT6, and ZT12, respectively (Multiple Comparison Tests: *p* = 0.002, *p* = 0.0001, *p* = 0.02, respectively).

To examine the direct exposure of light on the DLG level in the lamina, we examined DLG-specific immunofluorescence in flies after 15 or 60 min light pulses in DD (Figure 7D). The results revealed the significant influence of such stimulation on the DLG level in the lamina [Kruskal–Wallis Test: *H*_(2, *N* = 254)_ = 51.4, *p* < 0.0001]. A significant increase in the intensity of fluorescence (in comparison to the non-stimulated control group), was observed not only after the 60 min light pulse (21%), but also after the 15 min pulse (11%) (Multiple Comparison Test: *p* < 0.0001 for both comparisons). A significant difference (10%) in the level of fluorescence was also revealed between the two stimulated groups (Multiple Comparison Test: *p* = 0.004).

## Discussion

All three synaptic markers examined in our previous (Górska-Andrzejak et al., 2013) and present study showed both cyclic and stimuli-depended changes in their abundance in the lamina of *D. melanogaster*. This is the first report that the abundance of the synaptic structural proteins (BRP and DLG) and the proteins involved in synaptic vesicle release (SYN) is controlled by the circadian clock not only in neurons of the circadian clocks (Shapiro-Reznik et al., 2012) but also in other brain regions, such as the first optic neuropil (lamina). Moreover, the observed changes in the abundance of BRP, DLG, and SYN were specific for each protein. In contrast, no oscillations in these proteins, except BRP (Górska-Andrzejak et al., 2013), were detected in the whole head. Also their gene expression, except *dlg*, was not cycling when measured as the mRNA levels in the brain of flies sacrificed at different time points in the 24 h cycle. These results indicate that the expression of the synaptic proteins is specifically regulated in different regions of the brain.

Of the three synaptic proteins, BRP has already been studied in the brain and lamina of *D. melanogaster* and its abundance displayed both daily and circadian rhythms (Górska-Andrzejak et al., 2013). In the lamina the level of BRP increases twice during the 24 h cycle of LD 12:12; at the beginning of the day (ZT1) and at the onset of the night (ZT13). The same changes were observed in whole head homogenates analyzed using Western blotting. In DD the level of BRP increases only once during the 24 h cycle—at the beginning of the subjective night (CT13) (Górska-Andrzejak et al., 2013). The circadian rhythm of BRP is correlated with the circadian rhythm in the locomotor activity of *D. melanogaster* and the circadian plasticity of monopolar cells in the lamina (Pyza and Meinertzhagen, 1999; Weber et al., 2009).

In the present study we also found that the daily rhythm of BRP is not altered by locomotor stimulation. The stimulation, however, increases the level of BRP, especially at ZT1 and ZT13 when BRP normally peaks during the 24 h light/dark cycle. The level of BRP is also affected by light, since it increases after light pulses, and the circadian rhythm of BRP is entrained by light (Górska-Andrzejak et al., 2013). Moreover, among two peaks observed in BRP abundance during the day and night of LD 12:12, the morning peak is controlled by light but the evening one by the circadian clock, although both vanish in mutants with the null mutation of the clock gene *period* (*per*) (Górska-Andrzejak et al., 2013).

Our present results, as well as our previous findings (Górska-Andrzejak et al., 2013) indicate that BRP abundance at tetrad synapses is controlled by the circadian clock and modulated by light and other stimuli. Therefore, BRP organizes the tetrad presynaptic elements according to the activity of a fly and flow of visual and photic information from the eye photoreceptors.

The BRP level is also affected by sleep deprivation (Gilestro et al., 2009) and by activity stimulation, however, a short, 1 h locomotor stimulation does not change the daily pattern of BRP but magnifies the amplitude of the rhythm.

In the case of SYN the results are different than those obtained for BRP in LD 12:12 (Górska-Andrzejak et al., 2013). The level of SYN in the lamina, measured as SYN-specific immunofluorescence intensity, during the 24 h cycle of LD 12:12 did not change. There are also no significant changes in SYN level in the head analyzed using the Western blot technique. These results are surprising, since SYN, like BRP, constitutes presynaptic machinery. However, SYN is involved in the regulation of the reserve pool of vesicles (Akbergenova and Bykhovskaia, 2007), which is known to be released exclusively upon prolonged stimulation and high synaptic activity (Denker et al., 2011). During the normal activity of flies, the majority of these vesicles do not undergo either exocytosis, or recycling. It has been shown that during such circumstances only a surprisingly small percentage of vesicles (approximately 1–5%) maintains synaptic activity and is used repeatedly. Taking into account that normally active synapses appear not to require numerous reserve vesicles to sustain neurotransmitter release (Denker et al., 2011), it seems plausible that the level of SYN stays the same during the day and night. In DD on the other hand, the circadian rhythm was detected, with a higher level of SYN during the subjective day than during the subjective night. This reveals the influence of both the circadian clock and light on the expression of SYN. It also indicates that the circadian input increases the level of SYN synthesis during the subjective day. In LD 12:12 the circadian regulation seems to be masked by light, suggesting that the visual system is ready to recruit synaptic vesicles at any time during the day and night. In DD the SYN level was increased only by the short (15 min) light pulse and not by the long (60 min) one, which indicates the fast adaptation of SYN to light exposure.

SYN has been used, as a synaptic marker, in many studies in vertebrates and in *D. melanogaster*. The level of SYN increases during wakefulness and after sleep deprivation in the brain of *Drosophila* (Gilestro et al., 2009) and after exercise in the hippocampus and dentate gyrus of rats (Vaynman et al., 2004; Bechara et al., 2013). After locomotor stimulation, the increase in the SYN level in the lamina was observed only during the night. At the beginning of the day, the forced activity decreased the expression of SYN. This decrease may protect the visual system against overstimulation by light and the visual stimuli during high locomotor activity at the beginning of the day. This is also consistent with known data on SYN responses to intense stimulation. The SYN-dependent maintenance of synaptic release occurs under high-frequency nerve stimulation. The Ca^2+^ triggered increase in the phosphorylation of SYN, promotes recruitment of vesicles from the reserve pool to the active zone of the presynaptic element (Pieribone et al., 1995; Bloom et al., 2003; Menegon et al., 2006; Sun et al., 2006; Gaffield and Betz, 2007). In *synapsin*-null mutant of *D. melanogaster*, the proportion of vesicle recycling *in vivo* is increased by 30% (Denker et al., 2011).

Therefore, the two proteins, BRP and SYN, although both presynaptic and engaged in synaptic vesicle release during neurotransmission, appear to function in distinct modes that are activated upon low—or high—frequency stimulations (Knapek et al., 2011).

The third studied protein, DLG, belongs to the MAGUK family of proteins, which play a central role in molecular mechanisms involved in the regulation of spine morphogenesis and contact actin regulating proteins that are crucial for controlling the size and shape of dendritic spines (Zheng et al., 2011).

In *D.melanogaster* several transcripts are coded by the *dlg* gene, whilst two proteins, DLGA (PSD-95-like protein) and DLGS97 (SAP97-like protein) are main isoforms (Mendoza et al., 2003). The DLGA is expressed in the epithelia, somatic muscles and nervous system and the *dlga* mutation is lethal in the development (Woods and Bryant, 1991).

The *dlgs97* mutants survive until the adult stage but their behavior, including circadian rhythms, are disrupted (Mendoza-Topaz et al., 2008). In the present study we used the antibody which labels both DLGA and DLGS97 proteins, however, the observed changes are related to DLGS97 since this isoform predominates at the synapses in the brain of the adult flies (Mendoza-Topaz et al., 2008). In the lamina, we detected the circadian rhythm of DLG. Moreover, the pattern of the rhythm in DD was the same as in LD 12:12, with two morning/subjective morning and evening/subjective evening peaks. This pattern is characteristic for the daily rhythms in locomotor activity, plasticity of the lamina monopolar cells (Pyza and Meinertzhagen, 1999), activity of the sodium pump in the lamina (Górska-Andrzejak et al., 2009b; Damulewicz et al., 2013) and for the rhythm of BRP (Górska-Andrzejak et al., 2013).

We also found that *dlg* shows daily expression in the brain, which suggests that the cyclic expression of *dlg* might be crucial for rhythms in brain functions and in behavior. In *dlgS97* mutants, defects in phototaxis and courtship behavior, as well as disruption of the circadian rhythm in locomotor activity have been observed (Mendoza-Topaz et al., 2008). However, the rhythm in *dlg* expression was not maintained in constant darkness, so the circadian clock does not control its expression directly. Surprisingly the level of DLG, measured using Western blotting, does not oscillate in whole head homogenates. It may result from different rhythms of DLG isoforms or different patterns of DLG rhythm in various brain regions. In the lamina we observed DLG—immunofluorescence in pre- and postsynaptic cells but we were not able to examine pre- and postsynaptic changes in the abundance of DLG separately.

Having analyzed the daily rhythms of DLG and BRP, we can conclude that both scaffolding proteins organize proper synaptic contacts during high activity of the visual system and locomotor activity of flies. The correlation between activity and expression of DLG has also been found after sleep deprivation and long periods of wakefulness (Gilestro et al., 2009). In the first neuropil of the visual system we observed that the DLG level increases after light pulses in DD, activating the visual system but not after locomotor stimulation. The 1 h forced motor activity at four time points of LD 12:12 increased the abundance of DLG in the middle of the night. These results indicate that DLG, like SYN, may protect the visual system against overstimulation during the day by two stimuli, light, and locomotor activity, so DLG expression is suppressed during the day when active flies are additionally forced to fly.

Each of the three synaptic proteins that were studied appears to be endogenously regulated at synapses and additionally modulated by external stimuli. In case of the visual system, light is a strong stimulus that can synchronize, modulate, and/or mask the circadian expression of synaptic proteins. However, these proteins can also be modulated by non-visual stimuli, such as motor stimulation, which nonetheless have a stronger impact on the visual system than the visual stimulation (Kula and Pyza, 2007). In the lamina of *D. melanogaster*, light and motor stimulations not only affect synaptic protein expression but also the morphology of postsynaptic cells, which are remodeled in their structure during the day and night (Pyza and Meinertzhagen, 1995, 1999; Weber et al., 2009). The mechanisms of BRP, SYN, and DLG circadian regulation, and the effects of light and motor stimulations on their abundance are unknown. In our previous study on BRP we found that the circadian rhythm of this protein is abolished in the clock mutant *per*^0^ and its pattern is changed in another clock mutant *tim*^0^, in the circadian photoreceptor mutant *cry*^0^, and in the phototransduction mutant *norpA* (Górska-Andrzejak et al., 2013). These cyclic and stimuli-dependent changes of synaptic proteins may be responsible for several daily and circadian rhythms in the morphology of neurons (Pyza and Meinertzhagen, 1995, 1999; Weber et al., 2009), glial cells (Pyza and Górska-Andrzejak, 2004), activity of ion pumps (Pyza et al., 2004; Górska-Andrzejak et al., 2009b; Damulewicz et al., 2013) and in other rhythms detected in the fly's lamina (Pyza and Górska-Andrzejak, 2008; Pyza, 2010).

### Conflict of interest statement

The authors declare that the research was conducted in the absence of any commercial or financial relationships that could be construed as a potential conflict of interest.

## References

[B1] Akbergenova Y. Bykhovskaia M. (2007). Synapsin maintains the reserve vesicle pool and spatial segregation of the recycling pool in *Drosophila* presynaptic boutons. Brain Res. 1178, 52–64 10.1016/j.brainres.2007.08.042 17904536

[B2] Bechara R. G. Lyne R. Kelly A. M. (2013). BDNF-stimulated intracellular signalling mechanisms underlie exercise-induced improvement in spatial memory in the male Wistar. Behav. Brain Res. [Epub ahead of print]. 10.1016/j.bbr.2013.11.015 24269499

[B3] Bloom O. Evergren E. Tomilin N. Kjaerulff O. Low P. Brodin L. (2003). Colocalization of synapsin and actin during synaptic vesicle recycling. J. Cell. Biol. 161, 737–747 10.1083/jcb.200212140 12756235 PMC2199372

[B5] Budnik V. Koh Y. H. Guan B. Hartmann B. Hough C. Woods D. (1996). Regulation of synapse structure and function by the *Drosophila* tumor suppressor gene *dlg*. Neuron 17, 627–640 10.1016/S0896-6273(00)80196-8 8893021 PMC4661176

[B6] Chen G. Li W. Zhang Q. S. Regulski M. Sinha N. Barditch J. (2008). Identification of synaptic targets of *Drosophila* Pumilio. PLoS Comput. Biol. 4:e1000026 10.1371/journal.pcbi.1000026 18463699 PMC2265480

[B7] Damulewicz M. Rosato E. Pyza E. (2013). Circadian regulation of the Na+/K+-ATPase alpha subunit in the visual system is mediated by the pacemaker and by retina photoreceptors in *Drosophila melanogaster*. PLoS ONE 8:e73690 10.1371/journal.pone.0073690 24040028 PMC3769360

[B8] Denker A. Bethani I. Kröhnert K. Körber C. Horstmann H. Wilhelm B. G. (2011). A small pool of vesicles maintains synaptic activity *in vivo*. Proc. Natl. Acad. Sci. U.S.A. 108, 17177–17182 10.1073/pnas.1112688108 21903928 PMC3193224

[B9] Evergren E. Benfenati F. Shupliakov O. (2007). The synapsin cycle: a view from the synaptic endocytic zone. J. Neurosci. Res. 85, 2648–2656 10.1002/jnr.21176 17455288

[B10] Fouquet E. Owald D. Wichmann C. Mertel S. Depner H. Dyba M. (2009). Maturation of active zone assembly by *Drosophila* Bruchpilot. J. Cell. Biol. 186, 129–145 10.1083/jcb.200812150 19596851 PMC2712991

[B11] Fujita S. C. Inoue H. Yoshioka T. Hotta Y. (1987). Quantitative tissue isolation from *Drosophila* freeze-dried in acetone. Biochem. J. 243, 97–104 3111462 10.1042/bj2430097 PMC1147819

[B12] Gaffield M. A. Betz W. J. (2007). Synaptic vesicle mobility in mouse motor nerve terminals with and without synapsin. J. Neurosci. 27, 13691–13700 10.1523/JNEUROSCI.3910-07.2007 18077680 PMC6673622

[B13] Gavin B. A. Arruda S. E. Dolph P. J. (2007). The role of carcinine in signaling at the *Drosophila* photoreceptor synapse. PLoS Genet. 3:e206 10.1371/journal.pgen.0030206 18069895 PMC2134947

[B14] Gilestro G. F. Tononi G. Cirelli C. (2009). Widespread changes in synaptic markers as a function of sleep and wakefulness in *Drosophila*. Science 324, 109–112 10.1126/science.1166673 19342593 PMC2715914

[B15] Górska-Andrzejak J. Makuch R. Stefan J. Görlich A. Semik D. Pyza E. (2013). Circadian expression of the presynaptic active zone protein bruchpilot in the lamina of *Drosophila melanogaster*. Dev. Neurobiol. 73, 14–26 10.1002/dneu.22032 22589214

[B16] Górska-Andrzejak J. Niañko E. Pyza E. (2009a). Circadian expression of the presynaptic active zone protein Bruchpilot in the lamina of *Drosophila melanogaster*. J. Neurogenet. 23, S38–S39 [Meeting Abstract: V21]. 22589214 10.1002/dneu.22032

[B17] Górska-Andrzejak J. Salvaterra P. M. Meinertzhagen I. A. Krzeptowski W. Görlich A. Pyza E. (2009b). Cyclical expression of Na+/K+-ATPase in the visual system of *Drosophila melanogaster*. J. Insect. Physiol. 55, 459–468 10.1016/j.jinsphys.2009.02.003 19428365 PMC2721802

[B18] Guan B. Hartmann B. Kho Y. H. Gorczyca M. Budnik V. (1996). The *Drosophila* tumor suppressor gene, dlg, is involved in structural plasticity at a glutamatergic synapse. Curr. Biol. 6, 695–706 10.1016/S0960-9822(09)00451-5 8793296 PMC4658212

[B19] Hamanaka Y. Meinertzhagen I. A. (2010). Immunocytochemical localization of synaptic proteins to photoreceptor synapses of *Drosophila melanogaster*. J. Comp. Neurol. 518, 1133–1155 10.1002/cne.22268 20127822 PMC4029604

[B20] Hardie R. C. (1987). Is histamine a neurotransmitter in insect photoreceptors? J. Comp. Physiol. A 161, 201–213 10.1007/BF00615241 2442380

[B21] Hilfiker S. Pieribone V. A. Czernik A. J. Kao H. T. Augustine G. J. Greengard P. (1999). Synapsins as regulators of neurotransmitter release. Philos. Trans. R. Soc. Lond. B. Biol. Sci. 28, 269–279 10.1098/rstb.1999.0378 10212475 PMC1692497

[B22] Hofbauer A. Ebel T. Waltenspiel B. Oswald P. Chen Y. C. Halder P. (2009). The Wuerzburg hybridoma library against *Drosophila* brain. J. Neurogenet. 23, 78–91 10.1080/01677060802471627 19132598

[B22a] Kittel R. J. Wichmann C. Rasse T. M. Fouquet W. Schmidt M. Schmid A. (2006). Bruchpilot promotes active zone assembly, Ca^2+^ channel clustering, and vesicle release. Science 19, 1051–1054 10.1126/science.1126308 16614170

[B23] Knapek S. Sigrist S. Tanimoto H. (2011). Bruchpilot, a synaptic active zone protein for anesthesia-resistant memory. J. Neurosci. 31, 3453–3458 10.1523/JNEUROSCI.2585-10.2011 21368057 PMC6623931

[B24] Kula E. Pyza E. (2007). Effects of locomotor stimulation and protein synthesis inhibition of circadian rhythms in size changes of L1 and L2 interneurons in the fly's visual system. Dev. Neurobiol. 67, 1433–1442 10.1002/dneu.20518 17497696

[B25] Lahey T. Gorczyca M. Jia X. X. Budnik V. (1994). The *Drosophila* tumor suppressor gene *dlg* is required for normal synaptic bouton structure. Neuron 13, 823–835 10.1016/0896-6273(94)90249-6 7946331 PMC4661177

[B26] Li L. Chin L. S. Shupliakov O. Brodin L. Sihra T. S. Hvalby O. (1995). Impairment of synaptic vesicle clustering and of synaptic transmission, and increased seizure propensity, in synapsin I-deficient mice. Proc. Natl. Acad. Sci. U.S.A. 92, 9235–9239 10.1073/pnas.92.20.9235 7568108 PMC40959

[B27] Meinertzhagen I. A. (1989). Fly photoreceptor synapses: their development, evolution, and plasticity. J. Neurobiol. 20, 276–294 10.1002/neu.480200503 2664074

[B28] Meinertzhagen I. A. Sorra K. E. (2001). Synaptic organization in the fly's optic lamina: few cells, many synapses and divergent microcircuits. Prog. Brain Res. 131, 53–69 10.1016/S0079-6123(01)31007-5 11420968

[B29] Melzig J. Burg M. Gruhn M. Pak W. L. Buchner E. (1998). Selective histamine uptake rescues photo- and mechanoreceptor function of histidine decarboxylase–deficient *Drosophila mutant*. J. Neurosci. 18, 7160–7166 9736639 10.1523/JNEUROSCI.18-18-07160.1998 PMC6793226

[B30] Mendoza C. Olguin P. Lafferte G. Thomas U. Ebitsch S. Gundelfinger E. D. (2003) Novel isoforms of Dlg are fundamental for neuronal development in *Drosophila*. J. Neurosci. 23, 2093–2101 [Correction (2007), 27]. 12657668 10.1523/JNEUROSCI.23-06-02093.2003 PMC6742002

[B31] Mendoza-Topaz C. Urra F. Barría R. Albornoz V. Ugalde D. Thomas U. (2008). DLGS97/SAP97 is developmentally upregulated and is required for complex adult behaviors and synapse morphology and function. J. Neurosci. 28, 304–314 10.1523/JNEUROSCI.4395-07.2008 18171947 PMC4656021

[B32] Menegon A. Bonanomi D. Albertinazzi C. Lotti F. Ferrari G. Kao H. T. (2006). Protein kinase A-mediated synapsin I phosphorylation is a central modulator of Ca^2+^-dependent synaptic activity. J. Neurosci. 26, 11670–11681 10.1523/JNEUROSCI.3321-06.2006 17093089 PMC6674776

[B33] Ohshiro T. Yagami T. Zhang C. Matsuzaki F. (2000). Role of cortical tumour-suppressor proteins in asymmetric division of *Drosophila* neuroblast. Nature 408, 593–596 10.1038/35046087 11117747

[B34] Parnas D. Haghighi A. P. Fetter R. D. Kim S. W. Goodman C. S. (2001). Regulation of postsynaptic structure and protein localization by the Rho-type guanine nucleotide exchange factor dPix. Neuron 32, 415–424 10.1016/S0896-6273(01)00485-8 11709153

[B35] Pieribone V. A. Shupliakov O. Brodin L. Hilfiker-Rothenfluh S. Czernik A. J. Greengard P. (1995). Distinct pools of synaptic vesicles in neurotransmitter release. Nature 375, 493–497 10.1038/375493a0 7777058

[B36] Pyza E. (2010). Circadian rhythms in the fly's visual systems, in Encyclopedia of the Eye, Vol. 1, ed Darlene A. D. (Oxford: Academic Press), 302–311

[B37] Pyza E. Borycz J. Giebultowicz J. M. Meinertzhagen I. A. (2004). Involvement of V-ATPase in the regulation of cell size in the fly's visual system. J. Insect. Physiol. 50, 985–994 10.1016/j.jinsphys.2004.08.003 15607501

[B38] Pyza E. Górska-Andrzejak J. (2004). Involvement of glial cells in rhythmic size changes in neurons of the housefly's visual system. J. Neurobiol. 59, 205–215 10.1002/neu.10307 15085538

[B39] Pyza E. Górska-Andrzejak J. (2008). External and internal inputs affecting plasticity of dendrites and axons of the fly's neurons. Acta Neurobiol. Exp. (Wars). 68, 322–333 18511964 10.55782/ane-2008-1698

[B40] Pyza E. Meinertzhagen I. A. (1993). Daily and circadian rhythms of synaptic frequency in the first visual neuropile of the housefly's (*Musca domestica* L.) optic lobe. Proc. Biol. Sci. 254, 97–105 10.1098/rspb.1993.0133 8290615

[B41] Pyza E. Meinertzhagen I. A. (1995). Monopolar cell axons in the first optic neuropil of the housefly, Musca domestica L., undergo daily fluctuations in diameter that have a circadian basis. J. Neurosci. 15, 407–418 7823145 10.1523/JNEUROSCI.15-01-00407.1995 PMC6578271

[B42] Pyza E. Meinertzhagen I. A. (1996). Neurotransmitters regulate rhythmic size changes amongst cells in the fly's optic lobe. J. Comp. Physiol. A 178, 33–45 10.1007/BF00189588 8568723

[B43] Pyza E. Meinertzhagen I. A. (1999). Daily rhythmic changes of cell size and shape in the first optic neuropil in *Drosophila melanogaster*. J. Neurobiol. 40, 77–88 10398073 10.1002/(sici)1097-4695(199907)40:1<77::aid-neu7>3.0.co;2-0

[B44] Rogero O. Hammerle B. Tejedor F. J. (1997). Diverse expression and distribution of Shaker potassium channels during the development of the *Drosophila* nervous system. J. Neurosci. 17, 5108–5118 9185548 10.1523/JNEUROSCI.17-13-05108.1997 PMC6573291

[B45] Ruiz-Canada C. Koh Y. H. Budnik V. Tejedor F. J. (2002). DLG differentially localizes Shaker K^+^-channels in the central nervous system and retina of *Drosophila*. J. Neurochem. 82, 1490–1501 10.1046/j.1471-4159.2002.01092.x 12354297

[B46] Shapiro-Reznik M. Jilg A. Lerner H. Earnest D. J. Zisapel N. (2012). Diurnal rhythms in neurexins transcripts and inhibitory/excitatory synapse scaffold protein in the biological clock. PLoS ONE 7:e37894 10.1371/journal.pone.0037894 22662246 PMC3360661

[B47] Südhof T. C. (2004). The synaptic vesicle cycle. Annu. Rev. Neurosci. 27, 509–547 10.1146/annurev.neuro.26.041002.131412 15217342

[B48] Sun J. Bronk P. Liu X. Han W. Südhof T. C. (2006). Synapsins regulate use-dependent synaptic plasticity in the calyx of Held by a Ca^2+^/calmodulin-dependent pathway. Proc. Natl. Acad. Sci. U.S.A. 103, 2880–2885 10.1073/pnas.0511300103 16481620 PMC1413856

[B49] Tejedor F. J. Bokhari A. Rogero O. Gorczyca M. Zhang J. Kim E. (1997) Essential role for *dlg* in synaptic clustering of Shaker K^+^ channels *in vivo*. J. Neurosci. 17, 152–159 8987744 10.1523/JNEUROSCI.17-01-00152.1997 PMC4658234

[B50] Thomas U. Kim E. Kuhlendahl S. Koh Y. H. Gundelfinger E. D. Sheng M. (1997) Synaptic clustering of the cell adhesion molecule fasciclin II by discs-large and its role in the regulation of presynaptic structure. Neuron 19, 787–799 10.1016/S0896-6273(00)80961-7 9354326 PMC4658217

[B51] Vaynman S. Ying Z. Gomez-Pinilla F. (2004). Hippocampal BDNF mediates the efficacy of exercise on synaptic plasticity and cognition. Eur. J. Neurosci. 20, 2580–2590 10.1111/j.1460-9568.2004.03720.x 15548201

[B52] Wagh D. A. Rasse T. M. Asan E. Hofbauer A. Schwenkert I. Dürrbeck H. (2006). Bruchpilot, a protein with homology to ELKS/CAST, is required for structural integrity and function of synaptic active zones in *Drosophila*. Neuron 49, 833–844 10.1016/j.neuron.2006.02.008 16543132

[B53] Wang S. Yang J. Tsai J. Kuca T. Sanny J. Lee J. (2011). *Drosophila* adducin regulates Dlg phosphorylation and targeting of Dlg to the synapse and epithelial membrane. Dev. Biol. 357, 392–403 10.1016/j.ydbio.2011.07.010 21791202

[B54] Weber P. Kula-Eversole E. Pyza E. (2009). Circadian control of dendrite morphology in the visual system of *Drosophila melanogaster*. PLoS ONE 4:e4290 10.1371/journal.pone.0004290 19173003 PMC2628732

[B55] Woods D. F. Bryant P. J. (1991). The discs-large tumor suppressor gene of *Drosophila* encodes a guanylate kinase homolog localized at septate junctions. Cell 66, 451–464 10.1016/0092-8674(81)90009-X 1651169

[B56] Woods D. F. Hough C. Peel D. Callaini G. Bryant P. J. (1996). Dlg protein is required for junction structure, cell polarity, and proliferation control in *Drosophila* epithelia. J. Cell. Biol. 134, 1469–1482 10.1083/jcb.134.6.1469 8830775 PMC2120992

[B57] Zheng C. Y. Seabold G. K. Horak M. Petralia R. S. (2011). MAGUKs, synaptic development, and synaptic plasticity. Neuroscientist 17, 493–512 10.1177/1073858410386384 21498811 PMC3191319

